# Robot-Assisted Laparoscopic Nephroureterectomy versus Hand-Assisted Laparoscopic Nephroureterectomy for Upper Urinary Tract Urothelial Carcinoma: A Matched Comparison Study

**DOI:** 10.1155/2015/918486

**Published:** 2015-10-11

**Authors:** Che-Yuan Hu, Cheng-Kuang Yang, Chao-Yuan Huang, Yen-Chuan Ou, Shun-Fa Hung, Shiu-Dong Chung, Yeong-Shiau Pu

**Affiliations:** ^1^National Taiwan University Hospital, No. 7, Chung Shan S. Road (Zhongshan S. Road), Zhongzheng District, Taipei City 10002, Taiwan; ^2^Taichung Veterans General Hospital, Taiwan Boulevard, Sect. 4, Taichung 40705, Taiwan; ^3^Far Eastern Memorial Hospital, No. 21, Sec. 2, Nanya S. Road, Banqiao District, New Taipei City 220, Taiwan

## Abstract

*Objectives*. To investigate the perioperative and oncological outcomes of hand-assisted laparoscopic nephroureterectomy (HANU) and robotic-assisted nephroureterectomy (RANU). *Methods*. Patients who underwent RANU were matched by sex, age (±5 years), and tumor location to those who underwent HANU; 18 matched pairs were included. *Results*. Each group consisted of five men and 13 women. The mean age was 70.4 years in RANU group and 69.6 years in HANU group (*p* = 0.646). Each group contained 10 patients with tumor location in the renal pelvis, five in the ureter, and three in both sites. The median follow-up time was 6.1 months for the RANU group and 47.8 months for the HANU group. The demographic and pathological data did not differ significantly. The RANU group had significantly less blood loss (*p* < 0.001), resumed oral intake earlier (*p* = 0.043), and had shorter hospital stays (*p* = 0.014) but higher pain scores associated with their wounds (*p* = 0.043). The oncological outcomes were comparable with those of the HANU group. *Conclusions*. Our results show that the RANU and HANU groups have comparable operative, early postoperative, and functional outcomes. A longer follow-up period would be needed for final comparison of oncological outcome.

## 1. Introduction

Since laparoscopic nephroureterectomy (LNU) was introduced in 1991 by Clayman et al. [1], it has lowered postoperative morbidity rates and shortened hospital stays compared with open nephroureterectomy [2]. The drawbacks of the laparoscopic approach are longer operative time and the requirements for high levels of laparoscopic skill, since intracorporal suturing of the bladder is often needed after bladder cuff resection. Additionally, when the tumor is located in the distal third of the ureter, it often takes considerable effort to perform pure LNU, due to the increased difficulty of bladder cuff management. In contrast, hand-assisted laparoscopic nephroureterectomy (HANU) allows the surgeon to keep one hand within the body. This approach affords the use of tactile sensation, blunt manual dissection, and broad retraction. It also decreases operative time and allows surgeons to perform minimally invasive procedures for larger and more extensive tumors [3, 4]. Since the incidence of upper tract urothelial carcinoma (UTUC) is relatively high (about 31.4%) in Taiwan [5] compared to all other genitourinary malignancies (about 5% for the world) [6], HANU is more often used to reduce operative time and get better control of tumors.

In 2006, the first da Vinci Robot System (Intuitive Surgical, Sunnyvale, CA, USA) was put into operation in Taiwan. With advances in the use of robotic urologic surgery, a number of medical centers in Taiwan now have experience of robot-assisted nephroureterectomy (RANU) [7]. By reducing the difficulty of intracorporal suturing, RANU has the potential to make lower ureter and bladder cuff management easier than pure LNU. Nonetheless, the advantages and disadvantages of this surgery are not well documented, because of a lack of comparative studies between RANU and other minimally invasive techniques. We thus sought to compare the perioperative outcomes and short-term oncological outcomes of RANU and HANU.

## 2. Patients and Methods

The Institutional Review Board from National Taiwan University Hospital (NTUH) Research Ethics Committee (REC) approved this retrospective study and waived the informed consent requirement. From 2011 to 2013, eighteen UTUC patients who underwent RANU were exactly matched by sex, age (±5 years), and tumor location in a stepwise procedure to patients who underwent HANU from 2000 to 2013. Propensity score matching was not applied in this study. Excluding those that did not meet oncologic diagnosis or those who lacked sufficient data, a total of 197 patients who underwent HANU were eligible for matching. Eventually 18 matched pairs with UTUC were included in the study. Surgeons chose the operative method for UTUC patients during the time that RANU and HANU were both available. Each group consisted of five men and 13 women. In the RANU group, the patients were not repositioned after the nephrectomy. Nonetheless, the robot was redocked for excision of the distal ureter and bladder cuff.

We placed the patient in the lateral flank position with the diseased side up. A 12 mm camera port was then inserted in the periumbilical region and pneumoperitoneum was created. The first 8 mm robotic port was placed two fingers wide beneath the 12th rib, and the second 8 mm robotic port was introduced at the lateral edge of the rectus muscle, 3-4 cm below the umbilicus. A 12 mm assistant port was set up in the middle of the umbilicus and symphysis pubis, and a 5 mm assistant port was established in the middle of the umbilicus and xiphoid process.

**Figure 1:**
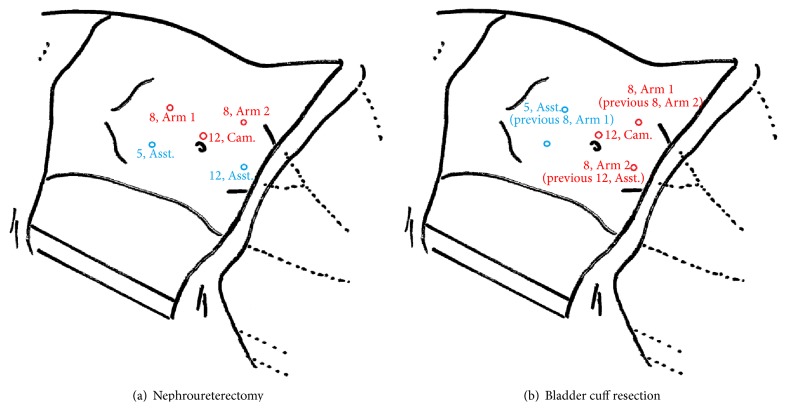
Robot-assisted laparoscopic nephroureterectomy in left-side, upper tract urothelial carcinoma patients; right-side in the symmetric configuration. 8, Arm 1: 8 mm working port for robotic arm 1; 8, Arm 2: 8 mm working port for robotic arm 2; 5, Asst.: 5 mm working port for assistant; 12, Asst.: 12 mm working port for assistant; 12, Cam.: 12 mm camera port.

We redocked the robot system for ureterectomy and bladder cuff resection after kidney dissection. The port for the first robotic arm became an assistant port. The port for the second robotic arm was converted to the port for the first robotic arm, and the assistant port was altered to the port for the second robotic arm [10]. We use this instrument configuration to dissect the distal ureter and bladder cuff, and intracorporeal suturing was performed to close the bladder wound (Figure 1). Finally, a 7 cm incision was made over lower abdominal midline near umbilicus for the specimen extraction.

In the HANU group, we made 7 cm Gibson's incision and created a site for the hand port, ureter identification and ligation, radical nephrectomy, and then distal ureter dissection. The remaining adventitial attachments to the bladder were identified by gentle traction on the ureter and dividing it with a dissector. Suturing of the bladder cuff and specimen extraction were accomplished through open Gibson's wound.

The follow-up schedule consisted of computed tomography examinations and bone scans at 6–12-month intervals or when clinically indicated. Cystoscopy was performed every 3 months in the first year, every 6 months in the next 2 years, and then annually. The grading and staging of the UTUC were performed according to the 1999 World Health Organization grade classification and the 2002 Tumor, Node, Metastasis Staging System. Clinical data, including patient survival rates and recurrence-free survival rate, bladder, renal pelvis, and ureter, were collected and analyzed. The Mann-Whitney *U* test, chi-square test, and Fisher's exact test were used for the statistical analysis. We used the Cox proportional hazard ratio (HR) model for the univariate analyses of oncological outcomes. In all of the tests, the statistical significance was set at *p* < 0.05.

## 3. Results

The median follow-up time was 6.1 months for the RANU group and 47.8 months for the HANU group. The median follow-up time for all 197 patients receiving HALNU was 41.8 months.  Table 1 shows the baseline characteristics of UTUC patients who underwent RANU or HANU.  Table 2 shows the clinical data from the matched cohort. The mean age of the RANU group was 70.4 years, while the mean age was 69.6 years in the HANU group. Both groups had similar clinical data, especially in terms of the previous abdominal operation history, hydronephrosis, ESRD, previous urine cytology, and simultaneous bladder cancer history.

Fifty percent or more of the tumors located in the renal pelvis in both the RANU and HANU groups (Table 3). Pathological TNM stage, grade, and lymphovascular invasion were also similar. One patient in the RANU group died from lung metastasis 6 months after surgery. The initial stage of her renal pelvic tumor was T1N0 and high grade. Three patients died of urothelial cancer in the follow-up period in the HANU group. One patient had bone metastasis (initial stage: T3N0, high grade; initial tumor location: renal pelvis and ureter) and one had recurrence in previous renal fossa (initial stage: T3N1, high grade; initial tumor location: ureter). The remaining patient had pancreatic and omental metastasis (initial stage: T3N0, high grade; initial tumor location: renal pelvis).

Table 4 provides the details of the perioperative outcomes. The mean operative time was 255.17 minutes in the RANU group and 250.17 minutes in the HANU group (*p* = 0.333). No patient complained of ileus after surgery. The mean blood loss, days to resuming oral intake, and length of hospital stay were significantly less in the RANU group than in the HANU group. Nonetheless, the pain scores of the associated wounds after surgery was significantly lower in the HANU group than in the RANU group.

The oncological outcomes are shown in Table 5. The relatively short follow-up time for the RANU group was due to the fact that this is a relatively new technology. Although no significant difference was noted in the oncological outcomes, two cases of recurrence in the renal fossa were noted in the HANU group.

## 4. Discussion

We compared the perioperative and oncological outcomes of two methods of modified LNU, RANU, and HANU. LNU provides good perioperative outcomes and cosmetic benefits [14]. HANU is used as an alternative, minimally invasive option with reliable cancer control for patients with advanced stage or tumor burdens. However, one recent study compared the outcomes of 722 patients who underwent pure LNU and 279 cases underwent HANU. It found that the use of the hand-assisted approach was associated with a higher bladder cancer recurrence rate (*p* < 0.01). The authors hypothesized that hand manipulation in the limited abdominal cavity may enhance tumor cell seeding and result in an increased intravesical recurrence rate [15]. RANU is a new technique that applies a magnified three-dimensional, highly precise vision system and tiny wristed instruments that bend and rotate like human wrists. Relatively few comparisons of the two techniques have been published showing the perioperative benefits and oncological outcomes of HANU and RANU.

In our study of these two techniques, most of the clinicopathological characteristics of the patients were very similar (previous abdominal operation history, hydronephrosis before surgery, urine cytology before surgery, ESRD before surgery, and simultaneous bladder cancer history), even though we matched only the age, sex, and tumor locations. Ambani et al. [16] conducted a matched comparison of RANU (*n* = 22) and LNU (*n* = 22). They found that the mean operative time (298 versus 251 minutes) and estimated blood loss (380 versus 233 mL) were significantly higher for RANU (*p* = 0.03 and *p* = 0.02, resp.). They proposed that the lack of experience with robotic surgery and robotic arm repositioning were the key factors influencing the prolonged operative time. They reported a higher rate of lymph node dissection (LND, 59% versus 27%) in the RANU group than in the LNU group, but the application of LND did not make a difference in operative time upon further analysis. Nevertheless, the application of LND was considered a possible explanation for the greater blood loss in the RANU group. The authors proposed that the features of magnified vision and the wristed instrumentation made the surgeons more confident to perform more extensive LND. However, due to the ambiguities regarding the benefits of LND during nephroureterectomy [17], we only performed LND if no significant complication or prolonged operative time was expected and whenever clinically indicated. In our study, the blood loss was 68.89 mL in the RANU group versus 358.33 mL in the HANU group (*p* < 0.001).

Ambani et al. placed their patients in the lithotomy position. They redocked the robotic system from the ipsilateral side of the patients' lesion to the side between their legs to complete the bladder cuff resection, whereas we kept the robotic system on the lateral side of the patients during surgery. The operative time did not significantly differ between the RANU and the HANU groups (255.17 versus 250.17 minutes, *p* = 0.333) in our study, in contrast to the findings of their earlier work. Park et al. [10] reported their initial hybrid-port technique for nephroureterectomy without redocking of the robot, and the total operative time was reduced by about 54 minutes compared to the method requiring relocation of the robot in between the patients' legs. The difference between the operative time in our study (255.17 minutes) and that of Ambani et al. (298 minutes) is about 43 minutes, which supports Park et al.'s findings.

In our study, no ileus or abdominal fullness sensation was noted after nephroureterectomy in either arm of the study, but the patients in the RANU group resumed oral intake earlier than those in the HANU group (*p* = 0.043). Additionally, the length of the hospital stay was shorter in the RANU group (*p* = 0.014). Despite these benefits, patients reported greater pain scores with RANU (*p* = 0.043). Since the total length of the surgical wounds was about the same (7 cm) in both groups after specimen retrieval, we thought this had little contribution to the difference in pain scores. Moreover, the oblique wound (Gibson's incision) across the lower quarter of the abdomen in the HANU group should make those patients feel more pain because of the transection of muscle and subcutaneous nerve in the abdominal wall. Due to the relatively crude movements of the robotic arm outside of the patients' bodies, more pressure may be applied on the cutaneous wound when patients were receiving RANU. This might explain the higher pain scores in the RANU group.

Table 6 lists the OP time, blood loss, hospital stay, and complications of patients receiving RANU in other study series. Most of the researches reported their initial experience of RANU, and the results varied from study to study. The blood loss was relatively low in our case, and we ascribe this to our previous practice with regard to other robotic-assisted urological surgeries [18], which helped us to perform RANU more proficiently.

The limitation of our study is the inherent bias of the retrospective design and small sample size. In addition, the follow-up period was relatively short. Furthermore, the surgeons' and patients' preferences determined the operation methods. Therefore, the statistical significance of the results should be interpreted with caution.

## 5. Conclusion

Our results show that RANU and HANU have comparable operative, early postoperative, and functional outcomes. The RANU group is superior with regard to blood loss, resumption of oral intake, and hospital stays, although higher pain scores associated with the wounds are noted. A longer follow-up would be needed for final comparison of oncological outcomes, although no difference is noticed in the present series.

## Figures and Tables

**Table 1:** Baseline characteristics of upper urinary tract urothelial carcinoma patients.

Variable	RANU	HANU	*p* value
	*n* = 18	*n* = 197	
Age (mean ± SD)	70.4 ± 6.3	67.7 ± 10.6	0.295
Gender			
Male	5 (27.8%)	104 (52.8%)	0.050^*∗*^
Female	13 (72.2%)	93 (47.2%)	
BMI (mean ± SD)	23.8 ± 3.4	24.6 ± 5.5	0.688
CKD stage			
Non-ESRD	15 (83.3%)	183 (92.9%)	0.159
ESRD	3 (16.7%)	14 (7.1%)	
Simultaneous bladder UC	4 (22.2%)	27 (13.7%)	0.303
Location			
Kidney	10 (55.6%)	117 (59.4%)	0.886
Ureter	5 (27.8%)	51 (25.9%)	
Both	3 (16.7%)	29 (14.7%)	
Tumor (T) stage			
Ta	3 (16.7%)	89 (45.4%)	0.052
T1	5 (27.8%)	30 (15.3%)	
T2	6 (33.3%)	58 (29.6%)	
T3	4 (22.2%)	16 (8.2%)	
T4	0	3 (1.5%)	
Node (N) stage			
N0	17 (94.4%)	175 (93.4%)	1.000
N1	1 (5.56%)	13 (6.6%)	
N2	0	6 (3.0%)	
N3	0	3 (1.5%)	
Metastasis (during surgery)	0	0	1.000
Grade			
Low	1 (5.56%)	24 (12.2%)	0.702
High	17 (94.4%)	173 (87.8%)	

HANU: hand-assisted laparoscopic nephroureterectomy; RANU: robot-assisted laparoscopic nephroureterectomy; BMI: body mass index; CKD: chronic kidney disease; UC: urothelial cancer. ^*∗*^ *p* < 0.05 indicates statistical significance.

**Table 2:** Clinical characteristics of upper urinary tract urothelial carcinoma patients.

Variable	RANU	HANU	*p* value
	*n* = 18	*n* = 18	
Age (mean ± SD)	70.4 ± 6.3	69.6 ± 5.7	0.646
Gender			
Male	5 (27.8%)	5 (27.8%)	1.000
Female	13 (72.2%)	13 (72.2%)	
BMI (mean ± SD)	23.8 ± 3.4	25.0 ± 4.9	0.411
Hydronephrosis	12 (66.7%)	11 (61.1%)	1.000
Previous abdominal operation	5 (27.8%)	4 (22.2%)	1.000
URS biopsy pathology			
Negative	6 (33.3%)	10 (55.6%)	0.186
Positive	12 (66.7%)	8 (44.4%)	
Urine cytology			
Negative	13 (72.2%)	14 (77.8%)	1.000
Positive	5 (27.8%)	4 (22.2%)	
CKD stage			
Non-ESRD	15 (83.3%)	16 (88.9%)	1.000
ESRD	3 (16.7%)	2 (11.1%)	
Simultaneous bladder UC	4 (22.2%)	4 (22.2%)	1.000
Herb use	1 (5.6%)	2 (11.1%)	0.486
ASA class			
I	0	0	0.051
II	8 (44.4%)	3 (16.7%)	
III	6 (33.3%)	10 (55.6%)	
IV	0	2 (11.1%)	

HANU: hand-assisted laparoscopic nephroureterectomy; RANU: robot-assisted laparoscopic nephroureterectomy; BMI: body mass index; URS: ureteroscopic; CKD: chronic kidney disease; ESRD: end-stage renal disease; UC: urothelial cancer; ASA: American Society of Anesthesiologists Physical Status.

**Table 3:** Pathological characteristics of upper urinary tract urothelial carcinoma patients.

Variable	RANU	HANU	*p* value
	*n* = 18	*n* = 18	
Location			
Kidney	10 (55.6%)	10 (55.6%)	1.000
Ureter	5 (27.8%)	5 (27.8%)	
Both	3 (16.7%)	3 (16.7%)	
Tumor (T) stage			
Ta	3 (16.7%)	7 (38.9%)	0.165
T1	5 (27.8%)	2 (11.1%)	
T2	6 (33.3%)	2 (11.1%)	
T3	4 (22.2%)	7 (38.9%)	
Node (N) stage			
N0	17 (94.4%)	16 (88.9%)	1.000
N1	1 (5.56%)	2 (11.1%)	
Metastasis (during surgery)	0	0	1.000
Grade			
Low	1 (5.56%)	3 (16.7%)	0.603
High	17 (94.4%)	15 (83.3%)	
Lymphovascular invasion	3 (16.7%)	4 (22.2%)	0.691

HANU: hand-assisted laparoscopic nephroureterectomy; RANU: robot-assisted laparoscopic nephroureterectomy.

**Table 4:** Perioperative outcome of hand-assisted laparoscopic nephroureterectomy (HANU) versus robot-assisted laparoscopic nephroureterectomy (RANU).

Variable	RANU	HANU	*p* value
	*n* = 18	*n* = 18	
Operative time, min (range)	255.17 (110–540)	250.17 (140–410)	0.333
Ileus	0	0	1.000
Blood loss, mL	68.89 (10–350)	358.33 (50–2000)	<0.001^*∗*^
Resumption of oral intake, days after NU	1.59 (0.5–3)	2.17 (1–3)	0.043^*∗*^
Hospital stay, days	6.79 (3.7–12)	9.61 (4–26)	0.014^*∗*^
Pain score, Visual Analog Scale of 1–10	6.22 (3–10)	3.93 (3–6)	0.043^*∗*^

^*∗*^ *p* < 0.05 indicates statistical significance.

**Table 5:** Oncological outcomes of hand-assisted laparoscopic nephroureterectomy (HANU) versus robot-assisted laparoscopic nephroureterectomy (RANU).

Variable	RANU	HANU	*p* value
	*n* = 18	*n* = 18	
Median follow-up, months (range)	6.1 (0.6–30.3)	47.8 (11.9–156.5)	
Overall recurrence	6 (33.3%)	10 (55.6%)	0.720
Tumor recurrence in the bladder	2 (11.1%)	6 (33.3%)	0.849
Tumor recurrence in the renal fossa	0	2 (11.1%)	0.516
Tumor recurrence in the previous ureter site	0	0	1.000
Distant recurrence (metastasis)	4 (22.2%)	2 (11.1%)	0.093
Cancer-specific death	1 (5.6%)	3 (16.7%)	0.729
Overall death	2 (11.1%)	5 (27.8%)	0.781

**Table 6:** Other study series of patients receiving RANU.

Study	Characteristics	Perioperative outcomes
Nanigian et al. 2006 [8]	10 patients Laparoscopic NU and robotic assisted laparoscopic BCR	(1) Mean OP time: 264 min (2) Average hospital stay: 3 days

Hu et al. 2008 [9]	(1) Five patients Flank position for NU; lithotomy position for distal ureter resection and BCR (2) Four patients Flank position for NU and BCR	(1) Mean blood loss: 211 mL (2) Mean OP time: 303 min (3) Mean hospital stay: 2.3 days

Park et al. 2009 [10]	(1) Six patients Flank position for NU; lithotomy position for distal ureter resection and BCR (2) Five patients Flank position for NU and BCR	(1) Mean blood loss: 106.7 versus 270.0 mL (2) Mean OP time: 247.3 versus 193.0 min (3) Mean hospital stay: 7.0 versus 8.4 days (4) Complication: 0 versus 0

Eandi et al. 2010 [11]	11 patients Flank position for NU; lithotomy position for distal ureter resection and BCR	(1) Median blood loss: 200 mL (2) Median OP time: 326 min (3) Mean hospital stay: 4.7 days

Hemal et al. 2011 [12]	15 patients Flank position for NU and BCR	(1) Mean blood loss: 103 mL (2) Mean OP time: 184 min (3) Mean hospital stay: 2.7 days

Pugh et al. 2013 [13]	43 patients Flank position for NU and BCR	(1) Mean blood loss: 131 mL (2) Mean OP time: 247 min (3) Median hospital stay: 3 days

NU: nephroureterectomy; BCR: bladder cuff resection; OP: operation.

## Abbreviations

HANU: Hand-assisted laparoscopic nephroureterectomy
RANU: Robot-assisted laparoscopic nephroureterectomy
BMI: Body mass index
URS: Ureteroscopic
CKD: Chronic kidney disease
ESRD: End-stage renal disease
UTUC: Upper tract urothelial carcinoma
ASA: American Society of Anesthesiologists Physical Status
BCR: Bladder cuff resection
OP: Operation.
